# A Case of Biliary Tract Infection Caused by KPC-2-Producing *Kluyvera ascorbata*

**DOI:** 10.1155/2018/5745708

**Published:** 2018-04-11

**Authors:** Lu Wang, Ying Jing, Kaisheng Lai, Jingna An, Jiyong Yang

**Affiliations:** ^1^Department of Microbiology, Chinese PLA General Hospital, Beijing, China; ^2^Department of Clinical Laboratory, Lu'an People's Hospital, Lu'an, Anhui, China

## Abstract

*Kluyvera* spp. can cause various infections. However, carbapenemase-producing *Kluyvera* spp. has not been previously reported. We report a case of biliary tract infection caused by KPC-2-producing *K. ascorbata* in a 13-year-old female. To the best of our knowledge, this is the first report on infection caused by carbapenemase-producing *Kluyvera* spp.

## 1. Introduction

*Kluyvera* is a small, flagellated, motile gram-negative bacillus in the family of Enterobacteriaceae, which has been identified in the natural environment, and is part of the normal flora of human gastrointestinal tract [1]. *Kluyvera* genus consists of four species: *K. ascorbata*, *K. cryocrescens*, *K. Georgiana*, and *K. cochleae*. *K. ascorbata* can cause various infections including urinary tract infection (UTI), bacteremia, and severe sepsis [1–5]. Here, we report a case of biliary tract infection caused by KPC-2-producing *K. ascorbata*.

## 2. Case Report

The patient was a 13-year-old female with hepatoblastoma who underwent left third hepatectomy in August 2017. Two months after the operation, the patient was readmitted to the hospital for signs of progressive jaundice and fever. CT examination revealed the presence of prehepatic fluid inclusion in the patient and, percutaneous transhepatic cholangial drainage (PTCD) under B-scan ultrasonography was subsequently performed. After the procedure, the patient showed persistent fever with leukocyte increase. On November 24, *K. ascorbata* was isolated from the biliary drainage. Levofloxacin in combination with meropenem was used to treat the infection. However, the patient's liver function continued to deteriorate with persistent fever. On December 10, the patient was discharged from the hospital for personal reasons.

The *K. ascorbata* isolate exhibited resistance to cephalosporins (ceftazidime, cefatriaxone, and cefepime) and carbapenems (imipenem and meropenem) with minimal inhibitory concentrations (MICs) of more than 64 mg/L and 16 mg/L, respectively. The carbapenem-resistant *K. ascorbata* isolate was screened for common carbapenemase genes (including *bla*_IMP_, *bla*_VIM_, *bla*_OXA-48_, *bla*_NDM_, and *bla*_KPC_) using PCR, and subsequent sequencing of amplicons was also performed. The *K. ascorbata* was confirmed to be a KPC-2-producing isolate.

## 3. Discussion

The *Kluyvera* genus was first named by a Dutch microbiologist Albert Jan Kluyver in 1936, and it consists of four species (*K. ascorbata*, *K. cryocrescens*, *K. Georgiana*, and *K. cochleae*). Since then, various infections caused by *Kluyvera* spp., including bacteremia, liver abscess, and urinary tract infection, have been reported worldwide [3]. In this case report, the patient suffered a biliary tract infection caused by *K. ascorbata*. Meropenem in combination with levofloxacin was used to treat the infection. However, the symptoms of the patient did not improve because of the resistance phenotype of the pathogen.

The *K. ascorbata* isolate from this patient was *bla*_KPC-2_ positive. As the most common carbapenemase, KPC-2 has been detected in *K. ascorbata* and *K. Georgiana* isolates from Israel and Brazil, respectively [6, 7]. Furthermore, polymyxin-resistance gene *mcr-1* has also been found in *K*. *ascorbata* [8]. The *Kluyvera* spp. has been commonly regarded as avirulent and could serve as a reservoir of resistance genes, leading to dissemination of these genes among different species and the spread of resistant pathogens among patients and in the environment [7]. For example, the resistant gene carried by *K. ascorbata* (named KLUA) has been confirmed to be the progenitor of CTX-M which is thought to be the most common and important extended-spectrum *β*-lactamase gene [9, 10]. The KLUA-producing *Kluyvera* spp. can survive for a long time in the environment, such as swage and the human gut, and promote drug resistance gene transfer [11]. The KPC-2-producing *K. ascorbata* has only been isolated from a rectal surveillance in Israel [7], and in this report, it was isolated from the specimen of infected site for the first time. Furthermore, combined use of antibiotics did not improve clinical symptoms of the infected patient, suggesting the tendency and possibility of resistant *Kluyvera* spp. as the pathogen of various infections rather than just a reservoir of resistance genes.

In conclusion, due to the characteristics of fast and easy transmission of various resistance genes, strict monitoring and more research are need for the control and successful treatment of drug-resistant bacterial infection and the prevention of the rapid transmission of the drug resistance genes.
